# The PB2, PA, HA, NP, and NS genes of a highly pathogenic avian influenza virus A/whooper swan/Mongolia/3/2005 (H5N1) are responsible for pathogenicity in ducks

**DOI:** 10.1186/1743-422X-10-45

**Published:** 2013-02-02

**Authors:** Masahiro Kajihara, Yoshihiro Sakoda, Kosuke Soda, Kenji Minari, Masatoshi Okamatsu, Ayato Takada, Hiroshi Kida

**Affiliations:** 1 Department of Disease Control, Graduate School of Veterinary Medicine, Hokkaido University, Sapporo, Hokkaido, 060-0818, Japan; 2 Research Center for Zoonosis Control, Hokkaido University, Sapporo, Hokkaido, 001-0020, Japan; 3 Present address: Division of Global Epidemiology, Research Center for Zoonosis Control, Hokkaido University, Sapporo, Hokkaido, 001-0020, Japan; 4 Present address: Avian Zoonosis Research Center, Faculty of Agriculture, Tottori University, Tottori, 680-8553, Japan

**Keywords:** H5N1 influenza virus, Duck, Natural host, Pathogenicity

## Abstract

**Background:**

Wild ducks are the natural hosts of influenza A viruses. Duck influenza, therefore, has been believed inapparent infection with influenza A viruses, including highly pathogenic avian influenza viruses (HPAIVs) in chickens. In fact, ducks experimentally infected with an HPAIV strain, A/Hong Kong/483/1997 (H5N1) (HK483), did not show any clinical signs. Another HPAIV strain, A/whooper swan/Mongolia/3/2005 (H5N1) (MON3) isolated from a dead swan, however, caused neurological dysfunction and death in ducks.

**Method:**

To understand the mechanism whereby MON3 shows high pathogenicity in ducks, HK483, MON3, and twenty-four reassortants generated between these two H5N1 viruses were compared for their pathogenicity in domestic ducks.

**Results:**

None of the ducks infected with MON3-based single-gene reassortants bearing the PB2, NP, or NS gene segment of HK483 died, and HK483-based single-gene reassortants bearing PB2, NP, or NS genes of MON3 were not pathogenic in ducks, suggesting that multiple gene segments contribute to the pathogenicity of MON3 in ducks. All the ducks infected with the reassortant bearing PB2, PA, HA, NP, and NS gene segments of MON3 died within five days post-inoculation, as did those infected with MON3. Each of the viruses was assessed for replication in ducks three days post-inoculation. MON3 and multi-gene reassortants pathogenic in ducks were recovered from all of the tissues examined and replicated with high titers in the brains and lungs.

**Conclusion:**

The present results indicate that multigenic factors are responsible for efficient replication of MON3 in ducks. In particular, virus growth in the brain might correlate with neurological dysfunction and the disease severity.

## Background

Influenza A viruses have eight-segmented, negative, and single-stranded RNA genomes and are serologically divided into 16 hemagglutinin (HA) (H1-H16) and 9 neuraminidase (NA) (N1-N9) subtypes [1,2]. Influenza A viruses are widely distributed in birds and mammals, including humans. Ecological studies have revealed that wild waterbirds, especially migratory ducks, are the natural hosts of influenza A viruses. Each of the known subtypes of influenza A virus has been perpetuated among water birds and in the water of the lakes where they nest in summer [3,4]. Furthermore, influenza A viruses circulating in nature are nonpathogenic in ducks and evolutionarily static [5,6], suggesting that the viruses and hosts have reached a long-established adaptive optimum.

Influenza A viruses maintained in ducks usually do not transmit to and infect chickens directly. It is known that low pathogenic viruses occasionally infect chickens after passage in domestic water birds such as ducks and geese and terrestrial birds such as quails and turkeys, and then may acquire high pathogenicity in chickens through multiple transmissions in the chicken population [7]. Highly pathogenic avian influenza viruses (HPAIVs) are so far restricted to H5 and H7 viruses, although most of the viruses of these subtypes are not highly pathogenic in chickens [8]. In 1997, outbreaks of highly pathogenic avian influenza (HPAI) occurred at live bird markets in Hong Kong and human cases of infection with the H5N1 virus were found [9,10]. Since then, H5N1 HPAIVs have thus been circulating in poultry for more than a decade [11].

It was generally thought that ducks could tolerate infection with influenza A viruses, including HPAIVs. In fact, A/Hong Kong/483/1997 (H5N1) (HK483) was not lethal for experimentally infected ducks [12]; however, in 2002, a large number of water birds, including ducks, geese, and other birds, died due to infection with H5N1 HPAIVs in Hong Kong [13]. In 2005, approximately six thousand migratory water birds were found dead with H5N1 virus infection in Qinghai Lake, China [14,15] and then virus strains of this subtype have been isolated in the Middle East, Europe, and Africa [16]. Since 2005, H5N1 HPAIVs originating from southern China have been isolated from dead aquatic birds such as swans and geese on their migratory routes to the northern nesting lakes in spring in Japan, Mongolia, and Russia [17-19]. It was reported that ducks experimentally infected with A/whooper swan/Mongolia/3/2005 (H5N1) (MON3) showed neurological dysfunction and died [17]. The mechanism whereby these H5N1 HPAIVs show high pathogenicity in ducks is still unclear.

In chickens, it is well known that insertion of a pair of dibasic amino acid residues at the cleavage site of the HA renders avian influenza viruses capable of infecting endothelial cells, followed by systemic infection [20-22]. Both HK483 and MON3 have multiple basic amino acid residues at the HA cleavage site, representing their high pathogenicity in chickens. The difference in pathogenicity in ducks between HK483 and MON3 hence suggests that factors other than the cleavage activation of the HA molecule contribute to the pathogenicity in ducks. To understand the molecular basis of the high pathogenicity of MON3 in ducks, reassortants were generated between HK483 and MON3 by reverse genetics and compared for their pathogenicity in domestic ducks. The present results indicate that multigenic factors were involved in the high pathogenicity of MON3 in ducks.

## Results

### Pathogenicity of H5N1 viruses in ducks of different ages

To investigate whether the age of ducks affects their susceptibility to H5N1 virus infection, either HK483 or MON3 was inoculated intranasally into 1-day-, 2-week-, and 4-week-old domestic ducks and clinical symptoms were monitored up to 14 days post-inoculation (dpi). None of the ducks inoculated with HK483 showed clinical signs for 14 days. On the other hand, all of the 1-day- and 2-week-old ducks inoculated with MON3 died by 3 and 7 dpi, respectively (Table 1). They showed depression and convulsion and some of the 2-week-old ducks showed torticollis. In 4-week-old ducks, two of the three birds infected with MON3 survived, although they showed depression, torticollis, and blindness. On 3 dpi, 1-day-, 2-week-, and 4-week-old ducks infected with either HK483 or MON3 were euthanized and lung and brain samples were collected to assess virus replication in ducks at different ages (Table 1). HK483 was recovered from each of the tissues tested from 1-day-old ducks; however, replication of HK483 was restricted in 2- and 4-week-old ducks; HK483 was not detected either in the brains of 2-week-old ducks or in the brains, tracheas, and livers of 4-week-old ducks (data not shown). On the other hand, MON3 was recovered from each of the tissues of ducks experimentally infected regardless of their age. As shown with high titers in the brains and lungs, both viruses replicated more efficiently in 1-day- and 2-week-old ducks than 4-week-old ducks (Table 1). The present data demonstrate that younger ducks were more susceptible to HK483 and MON3 than older ducks. In the following experiments, 2-week-old ducks were adopted, since obvious difference was found in mortality and virus replication in their brains between HK483 and MON3 infection.

**Table 1:** Comparison of pathogenicity of HK483 and MON3 in 1-day-, 2-week-, and 4-week-old ducks

**Virus**	**Age of ducks**	**Mortality**^**a**^	**MDD**^**b**^**(range)**	**Virus titers**^**c**^	
				**Brain**	**Lung**
HK483	1 day	0/3	≥ 14	5.4, 2.5, 2.5	5.5, 4.5, 2.5
	2 weeks	0/6	≥ 14	-, -, -^d^	4.3, 3.5, 2.5
	4 weeks	0/3	≥ 14	-, -, -	2.5, -, -
MON3	1 day	9/9	2.2 (2–3)	8.0, 7.5	5.7, 5.5
	2 weeks	10/10	4.9 (4–7)	8.8, 7.3, 7.3	8.5, 5.5, 5.3
	4 weeks	1/3	7.0	5.5, 4.5, -	4.5, 2.5, 3.7

^a^ Number of dead ducks/number of examined ducks.

^b^ Mean death days post-inoculation.

^c^ At 3 days post-inoculation, the brains and lungs were collected. Suspension of each tissue sample was inoculated into 10-day-old embryonated eggs for titration. Virus titers in tissues were expressed as log 50% egg infectious dose (EID_50_)/g. The lower limit of detection was 10^1.5^ EID_50_/g.

^d^ -: < 1.5 log EID_50_/g.

### Gene segments responsible for pathogenicity of MON3 in ducks

**Figure 1:**
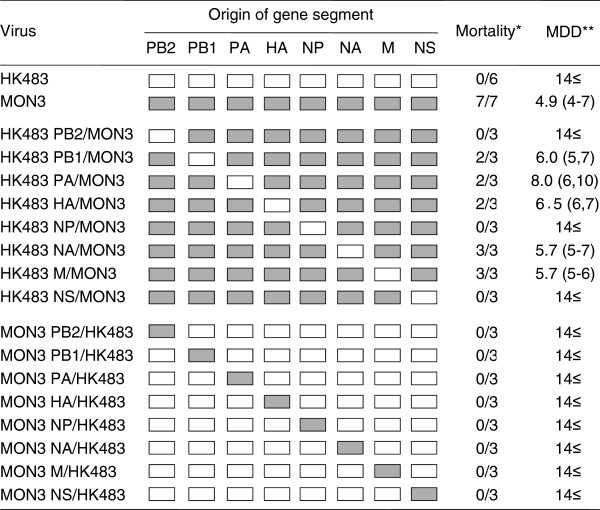
**Pathogenicity of HK483, MON3, and single-gene reassortants in 2-week-old ducks.** HK483, MON3, and a series of reassortants between HK483 and MON3 were generated by reverse genetics. Each of the viruses was inoculated into 2-week-old ducks and clinical symptoms of challenged ducks were observed over 14 dpi. White and gray boxes indicate the derivation of virus gene segments from HK483 and MON3, respectively. *: number of dead birds/number of examined ducks. **: mean death dpi.

In order to identify the gene segments responsible for the high pathogenicity of MON3 in ducks, MON3-based reassortants bearing a single segment of HK483 and the other seven segments of MON3 (HK483 PB2/MON3, HK483 PB1/MON3, HK483 PA/MON3, HK483 HA/MON3, HK483 NP/MON3, HK483 NA/MON3, HK483 M/MON3, and HK483 NS/MON3) were inoculated intranasally into three 2-week-old ducks to assess their pathogenicity. All ducks infected with HK483 NA/MON3 or HK483 M/MON3 showed severe depression and died on 5 to 7 dpi, as did ducks infected with MON3 (Figure 1). Pathogenicity of HK483 PB1/MON3, HK483 PA/MON3, and HK483 HA/MON3 in ducks was slightly lower than MON3 (i.e., one of three ducks infected with each reassortant survived for 14 days), although all ducks showed depression and the surviving duck infected with HK483 PB1/MON3 developed severe torticollis. HK483 PB2/MON3, HK483 NP/MON3, and HK483 NS/MON3 did not exhibit lethality in ducks, whereas some of the ducks infected with these reassortants showed lethargy and/or mild torticollis (Figure 1). Similarly, HK483-based reassortants bearing a single segment of MON3 and the other seven segments of HK483 (MON3 PB2/HK483, MON3 PB1/HK483, MON3 PA/HK483, MON3 HA/HK483, MON3 NP/HK483, MON3 NA/HK483, MON3 M/HK483, and MON3 NS/HK483) were inoculated into three 2-week-old ducks. Although some ducks showed mild lethargy, all the ducks survived for the 14-day observation period (Figure 1). These results revealed that the PB2, NP, and NS gene segments of MON3 were prerequisite for high pathogenicity in ducks; however, MON3 PB2/HK483, MON3 NP/HK483, and MON3 NS/HK483 did not cause severe clinical symptoms in any ducks, suggesting that multiple gene factors are involved in the high pathogenicity of MON3 in ducks.

**Figure 2:**
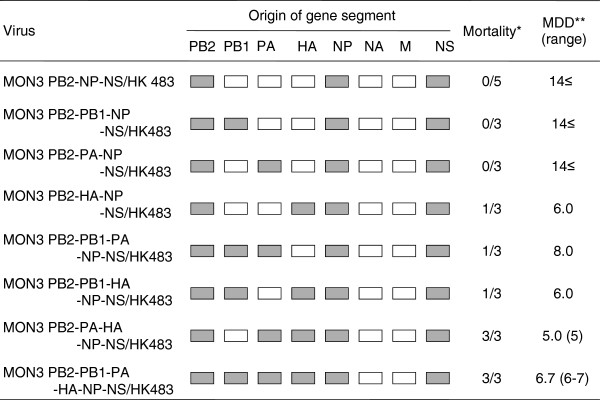
**Pathogenicity of multi-gene reassortants in 2-week-old ducks.** A series of multi-gene reassortants between HK483 and MON3 was generated by reverse genetics. Each of the viruses was inoculated into 2-week-old ducks and clinical symptoms of challenged ducks were observed over 14 dpi. White and gray boxes indicate the derivation of virus gene segments from HK483 and MON3, respectively. *: number of dead birds/number of examined ducks. **: mean death dpi.

To clarify the minimum set of MON3 gene segments required for its high pathogenicity in ducks, eight multi-gene reassortants between HK483 and MON3 were compared for their pathogenicity in ducks (Figure 2). These multi-gene reassortants uniformly possessed the MON3 PB2, NP, and NS gene segments prerequisite for high pathogenicity in ducks and the HK483 M and NA gene segments which unlikely contribute to the pathogenicity in ducks. These multi-gene reassortants had 8 possible combinations of the PB1, PA, and HA gene segments from HK483 and MON3 (Figure 2). These reassortants were designated MON3 PB2-NP-NS/HK483, MON3 PB2-PB1-NP-NS/HK483, MON3 PB2-PA-NP-NS/HK483, MON3 PB2-HA-NP-NS/HK483, MON3 PB2-PB1-PA-NP-NS/HK483, MON3 PB2-PB1-HA-NP-NS/HK483, MON3 PB2-PA-HA-NP-NS/HK483, and MON3 PB2-PB1-PA-HA-NP-NS/HK483. None of the ducks infected with MON3 PB2-NP-NS/HK483, MON3 PB2-PB1-NP-NS/HK483, or MON3 PB2-PA-NP-NS/HK483 manifested any clinical symptoms (Figure 2), except for a duck which showed lethargy upon MON3 PB2-NP-NS/HK483 infection. All the ducks inoculated with MON3 PB2-HA-NP-NS/HK483 showed depression and one of the three ducks died on 6 dpi. Following depression and cyanosis, all the ducks infected with MON3 PB2-PA-HA-NP-NS/HK483 died within 5 dpi as did ducks infected with MON3 (Figures 1 and 2). These results indicate that PB2, PA, HA, NP, and NS gene segments of MON3 were the required minimum set to show full pathogenicity in ducks.

### Replication of reassortants in 2-week-old ducks

**Figure 3:**
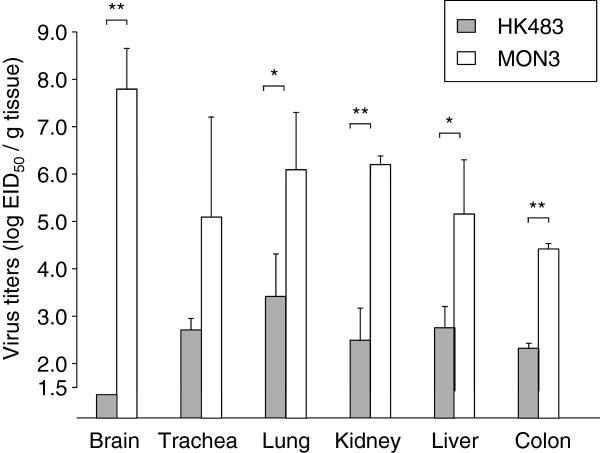
**Comparison of virus replication in 2-week-old ducks between HK483 and MON3.** Three days post-inoculation, the brains, tracheas, lungs, kidneys, livers, and colons were collected. Viruses in each of the tissue samples were titrated in 10-day-old embryonated chicken eggs and titers were expressed as EID_50_/g of tissues. The mean virus titers in each of the tissues and standard deviations are shown. The lower limit of detection was 10^1.5^ EID_50_/g. Virus titers were statistically analyzed by Student’s paired *t*-test. Significant differences are indicated by asterisks (** *p* < 0.001, * *p* < 0.05).

**Figure 4:**
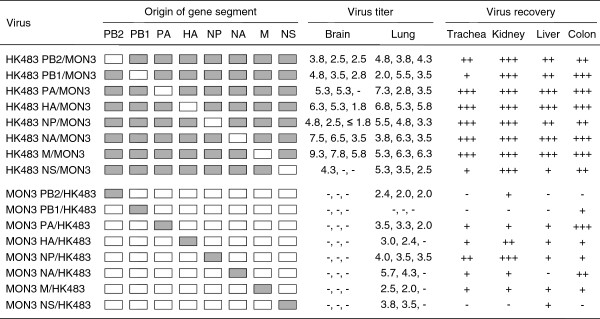
**Replication of single-gene reassortants in 2-week-old ducks.** A series of single-gene reassortants between HK483 and MON3 was generated by reverse genetics. Each of the viruses was inoculated into 2-week-old ducks and tissue samples were collected on 3 dpi. Suspension of each tissue sample was inoculated into 10-day-old embryonated eggs for virus titration or recovery. White and gray boxes indicate the derivation of virus gene segments from HK483 and MON3, respectively. Virus titers in tissues were expressed as log EID_50_/g and the lower limit of detection was 10^1.5^ EID_50_/g. +, ++, and +++: viruses were recovered from one, two, and three of three birds tested, respectively. -: viruses were not detected from tissue samples.

**Figure 5:**
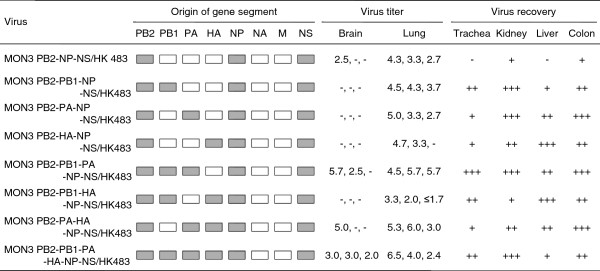
**Replication of multi-gene reassortants in 2-week-old ducks.** A series of multi-gene reassortants between HK483 and MON3 was generated by reverse genetics. Each of the viruses was inoculated into 2-week-old ducks and tissue samples were collected on 3 dpi. Suspension of each tissue sample was inoculated into 10-day-old embryonated eggs for virus titration or recovery. White and gray boxes indicate the derivation of virus gene segments from HK483 and MON3, respectively. Virus titers in tissues were expressed as log EID_50_/g and the lower limit of detection was 10^1.5^ EID_50_/g. +, ++, and +++: viruses were recovered from one, two, and three of three birds tested, respectively. -: viruses were not detected from tissue samples.

To further compare viral replication and distribution in ducks of HK483, MON3, and reassortants, virus titers in various tissue samples of ducks infected with these viruses were estimated on 3 dpi. Mean titers of MON3 were more than one hundred times as high as those of HK483 in each of the tissues tested (Figure 3). In particular, MON3 was detected at the highest titers in the brains among the tissues examined, while HK483 was not recovered from the brains. Dissemination of single- and multi-gene reassortants in 2-week-old ducks was also assessed on 3 dpi (Figures 4 and 5). Viruses that were lethal to ducks were recovered from various tissues and replicated with high titers especially in the brains. On the other hand, viruses that caused no disease in ducks replicated in restricted organs such as the lungs, kidneys, and colon. These data suggested that acute and efficient viral replication in systemic organs including the brain was the cause of death accompanying neurological symptoms.

## Discussion

Since the outbreaks of HPAI in waterbirds occurred in Hong Kong in 2002, a large number of free-flying water birds have died due to H5N1 HPAIV infection in the last decade; however, little is known about the mechanism whereby these viruses show high pathogenicity in ducks. It has been reported that tyrosine at position 436 of PB1 and threonine at position 516 of PA were associated with high pathogenicity in ducks [23]. Three amino acid residues in PB1-F2 were also reported as determinants of pathogenicity of an H5N1 HPAIV in mallards [24]. Recently, Song *et al.*[25] demonstrated that proline and aspartic acid at positions 224 and 383, respectively, of PA were involved in the virulence of H5N1 HPAIVs in domestic ducks. Here, we showed that the PB2, PA, HA, NP, and NS genes of MON3 were required to show full pathogenicity in ducks. The two strains utilized in the present study, HK483 and MON3, have comparably distant evolutionary relationship amongst H5N1 HPAIVs [17]. HA genes of HK483 and MON3 were classified into clade 0 and 2.2, respectively [17], and each gene segment of them possesses only 86.3–95.1% homology (Table 2). However, MON3 and HK483 share the same amino acid residues at all positions reported to be involved in the pathogenicity of H5N1 HPAIVs in ducks, suggesting other molecular determinants for the high pathogenicity of MON3 in ducks.

**Table 2:** Homology of viral genes and proteins between HK483 and MON3

	**Nucleotide (%)**	**Amino acid (%)**	**No. of different amino acids**
PB2	86.3	96.4	27
PB1	90.9		
PB1		97.2	21
PB1-F2		80.0	18
PA	88.5	95.3	34
HA	95.1	94.9	39
NP	92.1	97.6	12
NA	88.0	90.4	43
M	92.5		
M1		95.2	12
M2		95.9	4
NS	89.8		
NS1		85.2	34
NS2		90.1	12

The present study demonstrated that the PB2, NP, and NS genes of MON3 were prerequisite for high pathogenicity in ducks (Figures 1, 2, 4, and 5). Some explorations to clarify the relationship between the constellation of gene segments and pathogenicity of influenza A viruses were hitherto performed mainly using mammals, such as mice, ferrets, and monkeys [26-29]. These reports showed that the growth potential of reassortants differed depending on the constellation of the polymerase genes, suggesting the major contribution of the polymerases to efficient replication in mammals. NP was also shown to play an important role in the efficient replication and expansion of tissue tropism of H5N1 HPAIVs in chickens [30,31]. Recently, it was demonstrated that the interaction of PB2 and NP with importin-α, a host factor mediating trafficking into the nucleus, where transcription and replication of the viral genome occur, was correlated with host adaptation of influenza A viruses [32]. Amino acid differences of the PB2 and NP between HK483 and MON3 are 27 and 12 positions, respectively (Table 2). These differences are probably responsible for the incompatibility of the PB2 and NP of HK483 with viral proteins of MON3 to show high pathogenicity in ducks. Taken together, it is likely that the polymerase and the NP of MON3 may play a principal role in efficient replication in systemic organs of ducks coordinately with employing host factors involved in virus replication.

Sarmento *et al.*[33] investigated the contribution of the NS gene to the pathogenicity of H5N1 HPAIVs in ducks and discussed that the NS gene products had a minimal influence on the viral pathogenicity in ducks. Conversely, the present data showed that the NS gene segment of MON3 was also prerequisite for high pathogenicity in ducks (Figures 1 and 4). NS1, encoded in the NS gene, is well known to serve multiple functions in the life cycle of influenza A viruses [34]. The major role of NS1 is considered to be the inhibition of host innate immune responses by limiting interferon production and the subsequent antiviral effects of interferon-induced proteins [34]; therefore, NS1 has been extensively studied as a molecular determinant of viral pathogenicity mainly in mice and chickens. It was recently reported that deletion of amino acid residues 80–84 in NS1, which is a very common characteristic among H5N1 HPAIVs isolated after 2000, enhanced the pathogenicity of H5N1 viruses in mice and chickens [35]. It is noteworthy that MON3 also has the deletion of amino acid residues 80–84 in NS1 but HK483 does not; however, a plausible explanation of how NS1 with the deletion contributes to high pathogenicity is lacking and it is also unclear whether this finding is applicable to the pathogenicity of H5N1 viruses in ducks. Recently, Barber *et al.*[36] demonstrated that the cytoplasmic pathogen sensor RIG-I, triggering antiviral effects by recognition of viral RNA, might be associated with the natural resistance of ducks to influenza A virus infection, while RIG-I homologue of chickens has not been identified. It is known that the NS1 of some strains interacted with RIG-I and blocked its signaling pathway and subsequent interferon-β induction [34]; therefore, it is of interest whether the NS1 of MON3 suppresses innate immune responses of ducks more strongly than that of HK483.

Consistent with the data of Pantin-Jackwood *et al.*[37], the present study showed that the age of ducks is one of the factors which influenced their susceptibility to H5N1 HPAIVs. MON3 killed all of the 1-day- and 2-week-old ducks, but two of the three 4-week-old ducks infected with MON3 survived. While none of the ducks infected with HK483 died, this virus was recovered from the brains of 1-day-old ducks but not from the brains of 2- and 4-week-old ducks. As previously described by Kishida *et al.*[12], virus replication in the brain might be a cause of death upon HPAIV infection. Neurological symptoms, such as torticollis, blindness, and convulsion, were distinct characteristics of MON3 infection of ducks. MON3 showed considerably strong tropism for the central nervous system of 2-week-old ducks but HK483 were not detected in their brains (Figure 3), suggesting that efficient virus replication in the brain might correlate with neurological dysfunction and death. It is also known that chickens and humans die of multiple organ failure upon H5N1 virus infection [38,39]. In 2-week-old ducks, titers of MON3 were significantly higher than those of HK483 in all of the tissue tested (Figure 3). Furthermore, reassortants lethal to ducks showed wide tissue tropism and efficient replication in the lungs and brains (Figures 4 and 5); therefore, multiple organ failure also appeared to be critical for the high pathogenicity of MON3 in ducks.

The present study demonstrated that polygenic factors are involved in the high pathogenicity of MON3. In particular, the PB2, NP, and NS genes of MON3 were prerequisite. Emergence of H5N1 HPAIVs, which caused the death of the water birds in 2002, changed the idea that natural reservoir hosts (i.e., ducks) generally do not die due to influenza A virus infection. H5N1 HPAIVs that emerged in 1997 are still expanding their geographical distribution and the epidemiological study of HPAI in the 2010 migration season suggested the strong possibility that H5N1 HPAIVs might be perpetuated in a migratory bird population in the eastern Eurasian region [40]. These trends underscore the increasing importance of further study on avian influenza in migratory birds. To control HPAI not only in poultry but also in wild birds, the molecular basis of the high pathogenicity of H5N1 viruses in ducks should be clarified, in addition to continued global monitoring of HPAIVs in a migratory bird population.

## Conclusions

Experimental infection studies in ducks revealed that the PB2, NP, and NS gene segments of MON3 were prerequisite for the high pathogenicity of MON3 in ducks. A set of the PB2, PA, HA, NP, and NS gene segments of MON3 was required to show full pathogenicity in ducks. These data indicate that multigenic factors are responsible for the pathogenicity of MON3 in ducks. MON3 and reassortants that were lethal to ducks efficiently replicated in the tissues tested, especially in the brain, suggesting a possible correlation between virus growth in the brain and the death of ducks accompanying neurological dysfunction.

## Methods

### Viruses and cells

HK483 was isolated from throat aspirates of the third case of a 13-year-old girl. MON3 was isolated from the brain of a dead whooper swan found at Lake Khunt, Mongolia [17]. Viruses were propagated in 10-day-old embryonated chicken eggs for 48 hours at 35°C. Madin-Darby canine kidney (MDCK) cells and human embryonic kidney 293 T (293 T) cells were cultured according to Tsuda *et al.*[41].

### Generation of reassortants by reverse genetics

Viral RNAs of HK483 and MON3 were extracted as described previously [41]. Full-length genomes of the eight gene segments were cloned into a dual-promoter plasmid, pHW2000 [42]. HK483, MON3, and 24 reassortants between HK483 and MON3 were generated by reverse genetics (Figures 1 and 2). Briefly, MDCK and 293 T cells were cocultured in Opti-MEM 1 (Invitrogen) and transfected with 1 μg of each of the eight plasmids using TransIT-293 (Mirus Bio) according to the manufacturer’s protocol. After six hours of incubation at 37°C, transfection mixture was replaced with Opti-MEM 1. At 72 hours post-transfection, culture supernatant was collected and injected into the allantoic cavity of 10-day-old chicken embryonated eggs. Virus titers in harvested allantoic fluids were determined by the method of Reed and Muench [43] using embryonated eggs and expressed as EID_50_ per milliliter.

### Experimental infection of ducks

One-day-, 2-week-, and 4-week-old domestic ducks (Cherry Valley strain) were used to assess the pathogenicity of viruses. Viruses (10^6.0^ EID_50_ in 100 μl of allantoic fluid) were inoculated intranasally into three to nine ducks and clinical signs were monitored at 24-hour intervals over 14 dpi. Undiluted virus stocks of the following seven reassortants were inoculated into ducks, because their titers in 100 μl allantoic fluids did not reach 10^6.0^ EID_50_ despite attempts to increase their titers through several passages in embryonated eggs (i.e., 10^4.7^, 10^5.5^, 10^5.4^, 10^4.5^, 10^4.8^, 10^5.0^, and 10^4.5^ EID_50_ were used for MON3 HA/HK483, MON3 NS/HK483, MON3 PB2-PA-NP-NS/HK483, MON3 PB2-HA-NP-NS/HK483, MON3 PB2-PB1-HA-NP-NS/HK483, MON3 PB2-PA-HA-NP-NS/HK483, and MON3 PB2-PB1-PA-HA-NP-NS/HK483, respectively). Ducks exhibiting severe disease signs were euthanized by intravenous injection of pentobarbital (Dainippon Sumitomo Pharma) and recorded as having died the next day. To examine virus replication, each of the reassortants was inoculated into two or three ducks as described above. On 3 dpi, ducks were euthanized and the brains and lungs of 1-day-old ducks and the brains, tracheas, lungs, kidneys, livers, and colons of 2- and 4-week-old ducks were collected aseptically. To make 10% suspensions in minimum essential medium (Nissui Pharmaceutical), the tissue samples were homogenized by a Multi-Beads Shocker (Yasui Kikai). Virus titers in these suspensions were determined by the same method as described above and expressed as EID_50_ per gram of tissue.

Experimental infection of ducks was carried out in the biosafety level 3 facilities at the Graduate School of Veterinary Medicine, Hokkaido University, Japan according to the Guidelines for Proper Conduct of Animal Experiments of the Science Council of Japan (http://www.scj.go.jp/ja/info/kohyo/pdf/kohyo-20-k16-2e.pdf). The protocol was approved by the Hokkaido University Animal Care and Use Committee (Permit Number: 08-0157).

## Competing interests

The authors declare that they have no competing interests.

## Authors’ contributions

MK drafted the manuscript. MK, KS, and KM carried out generation of reassortants by reverse genetics and experimental infection of ducks. YS, MO, AT, and HK participated in the coordination of the study. All authors read and approved the final manuscript.
